# The Association of Surgeons in Training (ASiT) Consensus Statement on Major Trauma Training in the UK

**DOI:** 10.1308/rcsann.2022.0151

**Published:** 2023-02-07

**Authors:** A Thaventhiran, G McKnight, JM Clements, E Barlow, V Pegna, G Dovell, D Nally, J Burke

**Affiliations:** ^1^ The Association of Surgeons in Training, Royal College of Surgeons of England, UK; ^2^ Queen Mary University of London, UK; ^3^ Maidstone and Tunbridge Wells NHS Trust, UK; ^4^ Institute of Naval Medicine, UK; ^5^ Cardiff and Vale University Health Board, UK; ^6^ Royal College of Surgeons of England Council, UK; ^7^ University Hospitals Sussex NHS Foundation Trust, UK; ^8^ Somerset NHS Foundation Trust, UK; ^9^ Oxford University Hospitals NHS Foundation Trust, UK; ^10^ The Leeds Teaching Hospitals NHS Trust, UK

**Keywords:** Medical education, Surgery, Surgical training, Trauma

## Abstract

**Introduction:**

Since the establishment of the Major Trauma Networks in 2012, it is estimated that an extra 1,600 lives have been saved across England. Although the delivery of trauma care has improved significantly, the provision of trauma training has not and remains fragmented. The Association of Surgeons in Training (ASiT), an independent organisation run by trainees, is dedicated to excellence in surgical training within the United Kingdom (UK) and Republic of Ireland (ROI). The aim of this study was to develop a consensus statement representing the views of the ASiT on the future of trauma surgery training.

**Methods:**

A modified nominal group technique was used in five stages: 1, scoping exercise; 2, virtual consultation; 3, nominal group consensus meeting; 4, virtual feedback from stakeholders; and 5, virtual confirmation by the ASiT Council. The design and reporting of the consensus followed best practice methodology for consensus research.

**Results:**

Overall, 62 participants gave 90 statements across stages 1–3. Eleven key themes were identified, all of which met the consensus of the ASiT Council. The key findings were widespread support for increased exposure to trauma for medical students and early surgical trainees as well as an increased use of simulation methods and improved focus on non-technical skills within trauma surgery.

**Conclusions:**

This study sets out the position of the ASiT on the future of trauma surgery training and how training in major trauma surgery in the UK and ROI could be improved.

## Introduction

The delivery of trauma care in the United Kingdom (UK) has changed dramatically following publication of the National Confidential Enquiry into Patient Outcomes and Death report entitled ‘Trauma: Who Cares?’.^1^ The report highlighted deficiencies in trauma outcomes within the UK compared with the rest of the world. This resulted in the establishment of a network-based, hub-and-spoke model of major trauma centres (MTC) along with numerous regional trauma units. It is estimated that between 2008 and 2017 the introduction of this trauma system has saved an extra 1,600 lives.^2^ Trauma centres build up a ‘corporate memory’ for delivery of first-rate care and trauma research, which continues to improve patient outcomes following trauma. Despite rapid advancements in the organisation and delivery of trauma care, the training pathway for trauma surgeons in the UK and Republic of Ireland (ROI) has evolved at a much slower pace, and there is a paucity of data to illustrate how trainees believe their training should improve in this specialist area.

The next generation of trauma surgeons must be able to manage the most complex of patients, including those requiring transfusion pre-hospital blood products and endovascular resuscitation, as well as make best use of point-of-care technology such as viscoelastic testing to deliver tailored care to the critically injured patient. Trauma surgeons must also be skilled in the human factors required to work alongside and, where appropriate, lead the multidisciplinary team to ensure the delivery of the best possible care.

Many trainees who are interested in a career in trauma undertake a high-quality fellowship abroad, often in countries with dedicated systems for training and higher rates of penetrating trauma such as South Africa and the United States (USA).^3,4^ These fellowships can be rewarding both professionally and personally; however, they do not necessarily train in the hub-and-spoke model of trauma care used in the UK, or necessarily address the complex postoperative physical and emotional rehabilitation that is so crucial to the trauma team leadership role.^5^

The Association of Surgeons in Training (ASiT) is an independent professional organisation that represents and promotes excellence in surgical training for the benefit of both surgeons in training and patients. The ASiT represents all ten surgical specialties at every grade and in every region of the UK and ROI. This study aimed to ascertain trainees’ views on the barriers to training in trauma surgery and how training can be improved by using the nominal group technique (NGT) to develop consensus recommendations.

## Methods

### Study design

This cross-sectional study was registered prospectively (https://osf.io/msnj5/?view_only = 6b92faa419ac4186bc1f0755d7aead07) and was deemed to not require National Health Service Research Ethics Committee review (http://www.hra-decisiontools.org.uk/ethics/). However, the ASiT Council, the recognised representative body for surgical training in the UK, approved this study.

The design and reporting of this consensus followed the best practice methodology for consensus research.^6^ A modified nominal group consensus was used to generate ideas among surgical trainees and their representatives within the UK and ROI. NGT is a structured process of idea generation, collation and ranking based on their importance and relevance published by Gallagher in 1993.^7^ It facilitates initial open idea generation at the individual level without prejudice or bias from other group members, before combining statements to be agreed on by the majority of the group. The modified NGT occurred in five stages: 1, scoping exercise; 2, virtual consultation; 3, nominal group consensus meeting; 4, virtual consensus from stakeholders; and 5, virtual consensus from the ASiT Council.

#### Scoping exercise

A dedicated presentation and discussion was held at the annual National Trauma Research and Innovation Collaborative (NaTRIC https://www.c4ts.qmul.ac.uk/natric/natric) meeting on 7 February 2020. NaTRIC is a collaboration of surgical and medical trainees across multiple specialities along with established academics with an interest in both trauma and training. An online survey (Google Docs^®^; Google Corp.) was piloted to gather initial feedback and to allow for refinement prior to the next stages. The three identified study questions were:

1. What is lacking in major trauma training in the UK?
2. What are the barriers to major trauma training in the UK?
3. How can we improve major trauma training in the UK?

#### Virtual consultation

In the days preceding the annual ASiT conference in February 2020 social media was used to engage the ASiT membership and broader surgical community, and to promote discussion on the topic of trauma surgery training in the UK. The link to the online survey was advertised. These data were used to refine the survey and topics for discussion for stage 3.

#### Modified nominal group consensus

At the ASiT conference on 6 March 2020 in Birmingham, a dedicated group discussion was held using a NGT.^8^ Verbal discussion was encouraged to stimulate new ideas; however, the data were collected using an anonymous online survey (Google Docs^®^) that was completed over the course of a structured group discussion. A mixture of quantitative and qualitative data were collected. All members of the nominal group were invited to take part in stage 4.

#### Virtual consensus from stakeholders

A thematic analysis was performed, generating key themes and statements. The nominal group members were then invited to respond to an online survey (using Google Forms^®^) to rate their agreement or disagreement with the statements.

#### Virtual consensus

Virtual consensus was obtained from the ASiT Council, comprising 65 trainees representing all surgical specialities and geographical areas of the UK and ROI.

### Data analysis

Quantitative data are presented as percentages of all included respondents, followed by the number. Statistical analysis of quantitative data was conducted using GraphPad Prism (version 8.0.0 for Mac, GraphPad Software).

Thematic analysis of qualitative data was conducted using NVivo 11 (QSR International Pty Ltd, 2015) according to the principles of framework analysis. Following the first three rounds of surveys, the data gathered were used to generate specific statements. Respondents from rounds 1–3 were then invited to rate their agreement or disagreement with these statements. These statements were then presented to the ASiT Council, with Council members asked to rate their agreement with each of the statements on a five-point Likert scale ranging from ‘strongly disagree’ to ‘strongly agree’.

Any statements with >75% agreement or disagreement were considered ‘strongly recommended’ or ‘strongly recommended against’ accordingly, and any statements with 50%–75% agreement or disagreement were considered ‘recommended’ or ‘recommended against’ as appropriate. These statements were then taken to represent the ASiT Consensus Statement on Major Trauma Surgery Training in the UK and ROI.

Cronbach’s alpha was used to assess internal validity and a reliability coefficient value >0.70 was considered acceptable.

## Results

### Demographics

A total of 90 statements were recorded from 62 participants across the first three rounds. Table 1 describes the demographic details of these participants. A high degree of internal consistency was demonstrated in stages 1 and 2 (α = 0.959) as well as in stage 3 (α = 0.977).

**Table 1:** Demographic details of participants throughout stages of the nominal group technique

Variable	Responses, % (*n*)	Round 1	Round 2	Round 3	Round 5
Gender	Male			62.9 (39)	41.5 (27)
	Female			32.3 (20)	58.5 (38)
	Undeclared			4.8 (3)	0
Military	Regular			3.2 (2)	3.1 (2)
	Reserve			0	0
	No			96.8 (60)	97.0 (63)
Speciality	General surgery	33.3 (3)		38.7 (24)	47.7 (31)
	Vascular	33.3 (3)		8.1 (5)	6.2 (4)
	Plastics	0		11.3 (7)	4.6 (3)
	Orthopaedics	0		27.4 (17)	6.2 (4)
	Neurosurgery	0		6.5 (4)	4.5 (3)
	Cardiothoracic	0		6.5 (4)	4.5 (3)
	OMFS	22.2 (2)		1.6 (1)	1.5 (1)
	Urology	0		0	3.1 (2)
	ENT	0		0	4.5 (3)
	Paediatrics	0		0	4.5 (3)
	Undeclared	11.1 (1)		0	12.3 (8)
Grade	Medical student	0	0	16.1 (10)	3.1 (2)
	FY1–2	0	25 (2)	21.0 (13)	3.1 (2)
	FY3	0	0	8.1 (5)	3.1 (2)
	CT1–2	11.1 (1)	50 (4)	30.6 (19)	15.4 (10)
	ST3–5	44.4 (4)	0	8.1 (5)	15.4 (10)
	ST6–8	22.2 (2)	12.5 (1)	9.7 (6)	27.7 (18)
	SAS	11.1 (1)	0	4.8 (3)	0
	Consultant	0	0	1.6 (1)	0
	Undeclared	11.1 (1)	12.5 (1)	0	21 (32.3)
Region of Training/ Work	East of England	0		3.2 (2)	3.1 (2)
	East Midlands	11.1 (1)		22.6 (14)	4.5 (3)
	West Midlands	11.1 (1)		4.8 (3)	1.5 (1)
	Kent, Surrey, Sussex	11.1 (1)		8.1 (5)	4.5 (3)
	NE Thames	0		4.8 (3)	1.5 (1)
	NW Thames	0		4.8 (3)	1.5 (1)
	SE Thames	0		0	1.5 (1)
	SW Thames	0		6.5 (4)	3.1 (2)
	Mersey	0		1.6 (1)	3.1 (2)
	Northern	0		0	3.1 (2)
	North West	0		4.8 (3)	6.2 (4)
	Northern Ireland	0		6.5 (4)	3.1 (2)
	Republic of Ireland	0		1.6 (1)	6.2 (4)
	Oxford	0		0	3.1 (2)
	Peninsula	22.2 (2)		9.7 (6)	3.1 (2)
	East Scotland	0		0	3.1 (2)
	North Scotland	0		1.6 (1)	1.5 (1)
	South East Scotland	0		0	1.5 (1)
	West Scotland	11.1 (1)		8.1 (5)	3.1 (2)
	Yorkshire	0		0	3.1 (2)
	Wales	11.1 (1)		4.8 (3)	3.1 (2)
	Wessex	0		1.6 (1)	4.6 (3)
	Undeclared	22.2 (2)		4.8 (3)	30.8 (20)
Total responses (*n*)		9	8	62	65

CT = core training; ENT = ear, nose and throat; FY = Foundation Year; NE = North East; NW = North West; OMFS = oral and maxillofacial surgery; SAS = staff grade and associate specialist; SE = South East; SW = South West

### Thematic analysis

The qualitative questions from rounds 1 to 3 generated a total of 90 statements. These were analysed independently by two researchers (AT and GM); 11 themes were identified and the statements were grouped into four key themes: ‘professionalisation of major trauma’, ‘barriers to training in major trauma’, ‘training requirements’ and ‘diversity and flexibility’. Table 2 summarises the thematic analysis, alongside an example quote.

**Table 2:** Summary of thematic analysis of qualitative answers

		Responses		
Key theme	Specific theme	*n*	%	Example
Professionalisation of major trauma	Lack of defined training pathway	13	16.46	No definitive pathway/training scheme (Responder 1)
	Recognition as subspeciality or special interest	12	15.19	Subspecialisation [should be] attached to trauma surgeons [to be] able to train, with rotations dedicated to trauma (Responder 18)
	Lack of detailed curriculum	8	10.13	No curriculum (Responder 16)
Barriers to training in major trauma	Regional variation in exposure	22	27.85	Lack of exposure at DGHs following major trauma network established (Responder 2)
	Lack of volume	10	12.66	The UK doesn’t have enough trauma to train a cohort with a formal subspecialty (Responder 50)
	Under-represented in undergraduate curriculum	9	11.39	Issue [with trauma training] is more systemic and needs [to be] addressed in medical school with more anatomy (Responder 28)
Training requirements	Need for training in other specialities	8	10.13	Shared training between specialities, visiting other theatres, appreciating the cavity others operate in (Responder 9)
	Opportunity/necessity to train abroad	7	8.86	Overseas experience should be encouraged as OOPE (Responder 41)
	Desire for increased use of simulation for training	7	8.86	More simulated training scenarios (Responder 46)
Diversity and flexibility	Need to increase options for less than full-time training	4	5.06	Expense and work–life balance – not family-friendly (Responder 37)
	Increase BAME role models	1	1.27	Involve more BAME representation (Responder 14)

BAME = Black and Minority Ethnic; DGH = district general hospital; OOPE = out of programme experience

**Figure 1:**
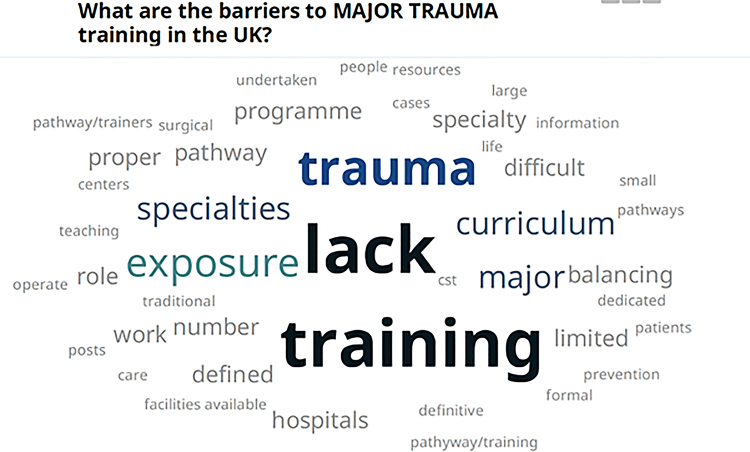
Word cloud representing the barriers to major trauma surgical training in the United Kingdom

**Figure 2:**
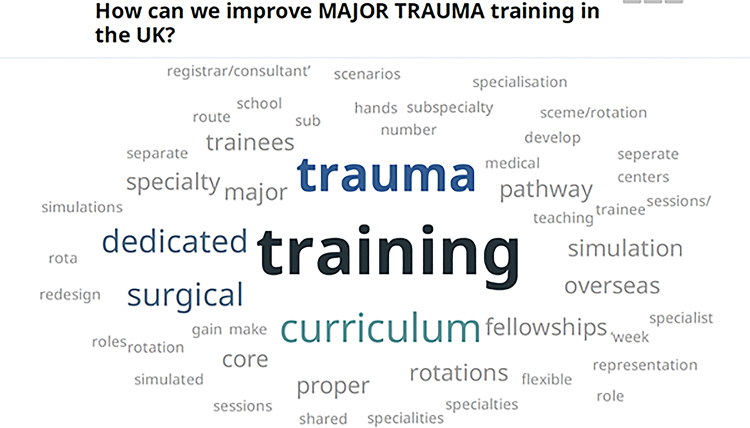
Word cloud representing the how major trauma surgical training in the United Kingdom and Republic of Ireland can be improved

The responses to the questions ‘What are the barriers to major trauma training in the UK and Ireland?’ and ‘How can we improve major trauma training in the UK and Ireland?’ are represented in word clouds in Figures 1 and 2.

### Professionalisation of major trauma

One of the most common themes throughout the thematic analysis was on the specialisation of major trauma. Thirteen (16.5%) respondents either called for a defined pathway for declared major trauma trainees or cited the lack of a recognised pathway as a barrier to those surgeons wishing to pursue a career in major trauma. A lower proportion of trainees surveyed (15.1%) desired the recognition of major trauma as a subspecialty and a further 10.1% stated that the lack of a defined curriculum was a training issue.

### Barriers to training

The most common barrier to training, reported by 27.85% (22) of respondents, was the regional variation of exposure during surgical training; this was reported to have been exacerbated by the creation of MTCs, which has led to a smaller number of trainees seeing trauma in their daily practice. Low trauma volume overall in UK surgical practice was also a concern; 12.66% (10) of respondents reported being concerned that they are unable to gain sufficient experience through UK surgical training. A smaller proportion (11.39%, 9) also reported concerns with the under-representation of major trauma within the undergraduate medical school curriculum and called for increased representation in this sphere to capture the attention of the future workforce.

### Training requirements

The most reported training requirement, described by 10.13% (8), was the need to train with other specialities outside the trainee’s base speciality: for example, general or vascular surgical training in cardiothoracic surgery or neurosurgery or vice versa. A further 8.86% (7) of trainees commented on their desire to undertake training abroad either as part of an Out of Programme Experience or a post-Certificate of Completion of Training (CCT) Fellowship. Countries with a higher burden of trauma than the UK were suggested as favourable fellowship destinations. The same number (8.86%, 7) of trainees also commented on the need for greater use of simulation within trauma surgery to improve training.

### Diversity and flexibility

Within this topic, there was a desire for more flexible training including options for Less Than Full-Time Training (LTFT) reported by 5.06% (4) of respondents. A smaller number, 1.2% (1), also called for more Black and Minority Ethnic role models within trauma surgery.

### Summary statements

On completion of the thematic analysis, a statement was written to summarise the theme and act as a recommendation to improve surgical training in major trauma in the UK and ROI. Respondents from rounds 1–3 were then asked to rate their agreement with the recommendations. All 11 statements were approved and taken forward for approval by the ASiT Council in round 5. The ASiT Council approved all 11. Each of these statements alongside their level of agreement from round 4 and 5 is shown in Table 3.

**Table 3:** Agreed statements with strength of recommendation from rounds 4 and 5

Number	Statement	Agreement from round 4	Agreement from round 5
1	The undergraduate curriculum should incorporate trauma surgery in order to increase visibility of the specialty and inspire the next generation of trauma surgeons.	Strongly recommended	Strongly recommended
2	An increase in placements for junior surgical trainees (CT or IST years 1 or 2) in major trauma centres (MTCs) and the introduction of optional taster weeks in the specialty for trainees without a dedicated trauma rotation.	Strongly recommended	Strongly recommended
3	Trauma competencies should be included in the Core Surgical curriculum including basic principles of haemorrhage control and specified procedures (both type and number).	Strongly recommended	Strongly recommended
4	Simulation and cadaveric training sessions should be introduced to the training programme to meet curriculum requirements and allow training in infrequent but essential procedures such as resuscitative thoracotomy and REBOA. This should be separate to optional courses such as those run by the royal colleges and other providers.	Strongly recommended	Strongly recommended
5	All higher speciality trainees (HSTs or registrars) should be offered a rotation in a major trauma centre (MTC).	Strongly recommended	Strongly recommended
6	Trauma surgery should be recognised as a declared special interest. This should be open to any surgeon with a suitable skillset, although this will most commonly be those with general or vascular surgery backgrounds.	Strongly recommended	Strongly recommended
7	The new Trauma Surgery section within the General Surgery curriculum should also include relevant leadership, management and human factors training.	Strongly recommended	Strongly recommended
8	Out of programme experiences (OOPE) should be encouraged to gain specialised training in trauma surgery. These OOPE could be either within a UK MTC or abroad, should be optional, and at the discretion of the trainee according to their own learning needs.	Strongly recommended	Strongly recommended
9	There should be an increase in exposure to specialities outside a trainee’s base speciality through increased flexibility in training placements. For example a general surgical trainee rotating through cardiothoracics or neurosurgery and vice versa.	Strongly recommended	Recommended
10	Trauma surgery should be recognised as a subspecialty within general and vascular surgery.	Recommended	Recommended
11	There should be a nationally coordinated competitive entry in to a trauma surgery training programme, following the Prehospital Emergency Medicine and trauma Training Interface Group (TiG) pathways. This should be open to anyone with a suitable skillset.	Recommended	Recommended

CT = core training; IST = improving surgical training; REBOA = resuscitative endovascular balloon occlusion of the aorta

## Discussion

This study highlights that major trauma is a thriving subspeciality encompassing both the difficult technical aspect and decision making–leadership skills that are attractive to many surgical trainees across a range of specialities. The 11 statements in Table 3 can be taken as clear recommendations from the ASiT on how surgical training in major trauma can be improved in the UK and ROI.

This is the first study based on the views of trainees to identify deficiencies in current surgical training, and to clearly recommend solutions that would attract high-calibre applicants to this growing subspeciality. Many trainees are attracted to the fast-paced nature of the work while operating in a multitude of different anatomical locations ‘… like the general surgeons of old’, as one trainee expressed. Others are attracted to the fact that emerging research is driving significant improvement in mortality, and the ability to work as part of a truly multidisciplinary team across a multitude of specialities and allied healthcare professions.^9,10^

### The new Intercollegiate Surgical Curriculum Programme curriculum

During the study period, the Intercollegiate Surgical Curriculum Programme (ISCP) moved to a new curriculum, with several key changes to the general surgery and vascular surgery curricula.^11^ One of the most significant changes was the recognition of ‘emergency general surgery and trauma’ as a defined subspecialty of general surgery. It contains all the syllabus requirements of the resuscitative surgeons pathway from the major trauma Training Interface Group (TiG).^12^ This syllabus allows trainees to attain the technical and leadership skills expected of a day 1 consultant major trauma surgeon at a UK MTC without the requirement for a post-CCT fellowship. Changes to the General Surgery ISCP Curriculum do provide a definitive pathway for general surgery trainees to follow; however, further work is required to ensure these competencies are achievable without the reliance on a post-CCT fellowship either abroad or as a UK-based TiG fellow. In addition, this new curriculum will not work in isolation; these competencies must be aligned, integrated and embedded in the training of the wider multidisciplinary team who contribute to the care of a trauma patient.

### Diversity and flexibility

One of the issues highlighted by trainees is the lack of ethnic and gender diversity within the speciality, although as the Royal College of Surgeons of England’s Kennedy Report highlighted that this is an issue across all surgical specialities.^13^ The royal colleges and the speciality associations need to publicise a list of diverse role models who can act as mentors and as a result reduce barriers currently experienced by trainees.

Finally, one major barrier trainees expressed is the perceived lack of ‘work–life balance’ and the speciality not being ‘family-friendly’. However, there are many inspiring role models who balance family life with surgical training, and surgical trainees are encouraged to seek LTFT training when appropriate.^14-16^ Burnout and the loss of surgical trainees to other specialities, alongside emigration abroad, is highlighted across all surgical specialities, with major trauma not singled out.^17-19^ The joint ASiT and British Orthopaedic Trainee Association LTFT study found over half of all surgical trainees, and a quarter of men, responded would consider LTFT.^20^

### Study limitations

Limitations of this study include the small number of participants and the subjective nature of the chosen methodology. However, the study used a validated methodology and was able to achieve a high level of internal consistency. Furthermore, as an independent organisation representing all specialities, all training grades and each region of the UK and ROI, the ASiT is well placed to synthesise the findings of the nominal group and is therefore in a position to provide a representative view of surgical trainees.

## Conclusions

This study is the first of its kind to explore the attitudes of surgical trainees towards a career in major trauma in the UK and ROI. It demonstrates that a wide variety of trainees are attracted to this fast-paced and challenging subspeciality and highlights the barriers that must be addressed to increase recruitment and retention into this field. The 11 statements outlined above can be taken as clear recommendations from the ASiT with regard to how trainees perceive the current training in trauma surgery and how this can be improved.
